# Host biology as a determinant of in vivo delivery and its translational potential

**DOI:** 10.1002/ctm2.70791

**Published:** 2026-08-24

**Authors:** Qirui Liang, Han Zhou, Yucai Wang

**Affiliations:** ^1^ State Key Laboratory of Immune Response and Immunotherapy Division of Life Sciences and Medicine Centre for Advanced Interdisciplinary Science and Biomedicine of IHM University of Science and Technology of China Hefei China

## 1 CLINICAL IMPORTANCE AND CURRENT LIMITATIONS OF IN VIVO DELIVERY SYSTEMS

In vivo delivery systems (IDSs) are essential for the development and clinical use of a wide range of therapeutics. Lipid nanoparticles enabled messenger RNA (mRNA) vaccines against coronavirus disease 2019 and respiratory syncytial virus,^1^ while liposomes, albumin nanoparticles, oncolytic viruses and adeno‐associated viral vectors have supported therapies for cancer and genetic diseases.^2^, ^3^ However, the clinical performance of many IDS‐based therapies remains limited by biological barriers after systemic administration, particularly rapid clearance by the mononuclear phagocyte system. The liver, especially Kupffer cells (KCs), captures a substantial fraction of administered IDSs before they reach diseased tissues.^4^ Efforts to overcome this barrier have therefore focused largely on carrier‐intrinsic properties, including particle size, surface chemistry, stealth modification and targeting ligand design.^5^ A recent study in Science, however, challenges this carrier‐centred view by showing that systemic clearance and therapeutic delivery are also actively regulated by endogenous host biology.^6^

## 2 CONCEPTUAL AND MECHANISTIC ADVANCES

**FIGURE 1:**
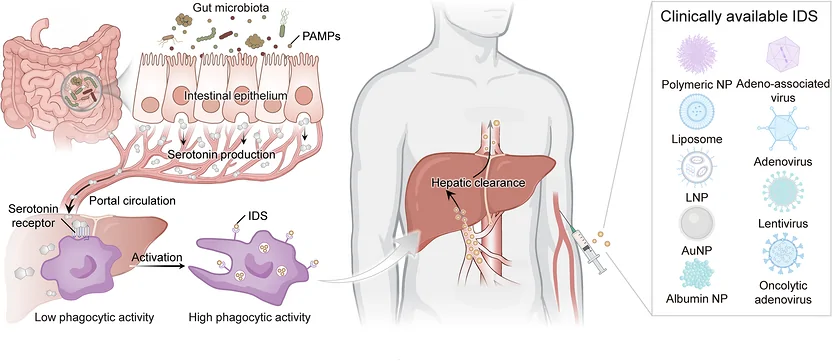
Inter‐organ regulation of hepatic clearance of in vivo delivery systems. Gut microbial signals are relayed through the intestinal epithelium and serotonin to regulate Kupffer cell (KC) phagocytic activity, thereby controlling hepatic sequestration of diverse synthetic and viral delivery systems. This gut–liver circuit illustrates how host physiology can regulate the systemic fate of therapeutic carriers.

A central conceptual advance of this study is the recognition that hepatic clearance is not an autonomous property of the liver. Rather, the clearance capacity of the liver can be actively programmed by inter‐organ communication and the broader physiological state of the host, thereby influencing the systemic fate and therapeutic performance of IDSs. Specifically, microbial signals from the gut are sensed by intestinal epithelial cells, which modulate serotonin production. Circulating serotonin then acts on KCs to enhance their phagocytic activity and promote the clearance of diverse synthetic and viral IDSs. These findings extend drug delivery beyond carrier engineering by showing that host biological pathways can also be targeted to modulate in vivo delivery (Figure 1).

A broader implication is that in vivo delivery can be improved not only by engineering the carrier, but also by modulating the host physiological programs that govern its clearance. This opens the possibility of targeting inter‐organ communication, metabolic signals, or immune‐cell states to control the in vivo fate of therapeutic carriers. Such host modulation may also expand the therapeutic use of modalities that are currently constrained by rapid systemic clearance, including oncolytic viruses. More broadly, delivery strategies may need to account for the physiological state of the individual patient, moving the field toward more personalised approaches to in vivo delivery.

## 3 CLINICAL AND TRANSLATIONAL IMPLICATIONS

### 3.1 Improving therapeutic index and target‐organ delivery

Reducing hepatic scavenging may directly improve the therapeutic index of systemically administered IDSs. For mRNA therapeutics, in vivo genome editing, viral‐vector therapies and cancer nanomedicines, limiting KC‐mediated clearance could prolong circulation,^7^ increase exposure of extrahepatic targets and potentially reduce the dose required for therapeutic activity, thereby reducing non‐productive hepatic sequestration and delivery‐associated toxicity.

### 3.2 Redistributing hepatic delivery toward therapeutic target cells

Hepatocytes are major therapeutic targets for many genetic, metabolic and infectious diseases. Yet high liver accumulation does not necessarily translate into effective hepatocyte delivery because substantial amounts of IDSs may be sequestered by KCs. Reducing KC capture could redistribute IDSs within the liver and improve access to hepatocytes and other therapeutic target cells, benefiting liver‐directed siRNA, mRNA and genome‐editing therapies for diseases such as transthyretin amyloidosis, inherited metabolic disorders and chronic hepatitis B.^8^

### 3.3 Exploiting the gut–liver axis as a therapeutic intervention

The gut–liver axis also provides multiple opportunities for therapeutic intervention. Pharmacological inhibition of serotonin signalling could transiently reduce KC phagocytic activity, while dietary modulation of tryptophan availability may offer a non‐pharmacological means of altering hepatic clearance. Targeting multiple serotonin receptors may further improve the efficacy or controllability of this approach. Such interventions could be applied around the time of dosing to create a transient delivery‐permissive state without permanently altering the carrier or host physiology.

### 3.4 Explaining interpatient variability in delivery

Diet, antibiotic exposure, gastrointestinal disease, liver function and concomitant medications may alter microbiome function or serotonin metabolism, thereby affecting hepatic uptake, systemic exposure and tissue distribution.^9^ Such variability may contribute to differences in therapeutic response and toxicity even with the same formulation and dose. Incorporating these host variables into pharmacokinetic and pharmacodynamic analyses may improve dose selection, response prediction and safety assessment.

### 3.5 Expanding host regulation beyond the gut–liver axis

The gut–liver axis is unlikely to be the only inter‐organ pathway regulating the in vivo fate of therapeutic carriers. Systemically administered IDSs interact with multiple organs and can activate immune, metabolic and neuroendocrine responses that may in turn alter vascular transport, macrophage activity, tissue sequestration, or intracellular processing. Such feedback may be particularly relevant to platforms that activate innate immunity, including mRNA formulations and viral vectors. Identifying additional inter‐organ regulatory circuits could reveal new host targets for improving delivery.

## 4 CHALLENGES AND FUTURE PERSPECTIVES

Several challenges remain before host‐regulated delivery can be translated broadly. Its relevance in humans and across different therapeutic platforms needs to be established. Moreover, serotonin is unlikely to be the only gut‐derived signal regulating hepatic clearance, and other microbial metabolites and inter‐organ metabolic axes may represent an unexplored layer of delivery control.^10^ Importantly, reducing clearance should not be an end in itself; the goal should be to define an optimal clearance state that improves target‐cell delivery without increasing off‐target exposure or toxicity. Ultimately, the value of host modulation will need to be demonstrated through meaningful therapeutic advances, including whether it can enable systemic delivery of modalities currently constrained by rapid clearance, such as intravenously administered oncolytic viruses.

## AUTHOR CONTRIBUTIONS

**Qirui Liang** and **Han Zhou**: contributed equally to this work and drafted the manuscript. **Yucai Wang** and **Qirui Liang**: prepared the figures. **Yucai Wang**: conceptualised and supervised the work and critically revised the manuscript. All authors reviewed and approved the final version of the manuscript.

## CONFLICT OF INTEREST STATEMENT

The authors declare no conflict of interest.

## ETHICS STATEMENT

Not applicable. This article does not involve any new studies with human participants or animal subjects.

## References

[1] Locci M , Pardi N . Immunological mechanisms of mRNA vaccines for infectious diseases. Nature. 2026;654(8120):892‐901. doi: 10.1038/s41586‐026‐10599‐0 42342874 10.1038/s41586-026-10599-0

[2] Wang Q , Zeng C , Chen Z , et al. Nanoparticles reach metastatic tumours via enhanced permeability of adjacent vessels. Nat Nanotechnol. 2026;21(6):880‐891. doi: 10.1038/s41565‐026‐02171‐8 42286217 10.1038/s41565-026-02171-8

[3] Wang Q , Liang Q , Dou J , et al. Breaking through the basement membrane barrier to improve nanotherapeutic delivery to tumours. Nat Nanotechnol. 2024;19(1):95‐105. doi: 10.1038/s41565‐023‐01498‐w 37709950 10.1038/s41565-023-01498-w

[4] de Lázaro I , Mooney DJ . Obstacles and opportunities in a forward vision for cancer nanomedicine. Nat Mater. 2021;20(11):1469‐1479. doi: 10.1038/s41563‐021‐01047‐7 34226688 10.1038/s41563-021-01047-7

[5] Cabral H , Li J , Miyata K , et al. Controlling the biodistribution and clearance of nanomedicines. Nat Rev Bioeng. 2024;2(3):214‐232. doi: 10.1038/s44222‐023‐00138‐1

[6] Wang Q , Chen Z , Zhang G , et al. Commensal‐driven serotonin production modulates in vivo delivery of synthetic and viral vectors. Science. 2026;391(6791):eadu7686. doi: 10.1126/science.adu7686 41855345 10.1126/science.adu7686

[7] Ouyang B , Poon W , Zhang YN , et al. The dose threshold for nanoparticle tumour delivery. Nat Mater. 2020;19(12):1362‐1371. doi: 10.1038/s41563‐020‐0755‐z 32778816 10.1038/s41563-020-0755-z

[8] Zhang H , Guo Y , Jiao J , et al. A hepatocyte‐targeting nanoparticle for enhanced hepatobiliary magnetic resonance imaging. Nat Biomed Eng. 2023;7(3):221‐235. doi: 10.1038/s41551‐022‐00975‐2 36536254 10.1038/s41551-022-00975-2

[9] Hsu CL , Schnabl B . The gut‐liver axis and gut microbiota in health and liver disease. Nat Rev Microbiol. 2023;21(11):719‐733. doi: 10.1038/s41579‐023‐00904‐3 37316582 10.1038/s41579-023-00904-3 PMC10794111

[10] Chang M , Wang Y , Ha JH , et al. A transferable gut microbiota‐bile acid pathway programs nanomedicine pharmacokinetics and therapeutic response. Nat Mater. 2026. doi: 10.1038/s41563‐026‐02690‐8 10.1038/s41563-026-02690-8 42538384

